# The putative oncotarget CSN5 controls a transcription-uncorrelated p53-mediated autophagy implicated in cancer cell survival under curcumin treatment

**DOI:** 10.18632/oncotarget.11940

**Published:** 2016-09-10

**Authors:** Qing-Yu Zhang, Rui Jin, Xian Zhang, Ji-Po Sheng, Fang Yu, Ren-Xiang Tan, Ying Pan, Jun-Jian Huang, Ling-Dong Kong

**Affiliations:** ^1^ State Key Laboratory of Pharmaceutical Biotechnology, School of Life Sciences, Nanjing University, Nanjing 210023, P. R. China; ^2^ Institute of Biotechnology, AMMS, Beijing 100850, P. R. China

**Keywords:** curcumin, CSN5, p53, autophagy, cancer cell-killing effect

## Abstract

Curcumin has shown promise as a safe and specific anticancer agent. The COP9 signalosome (CSN) component CSN5, a known specific target for curcumin, can control p53 stability by increasing its degradation through ubiquitin system. But the correlation of CSN5-controlled p53 to anticancer therapeutic effect of curcumin is currently unknown. Here we showed that CSN5-controlled p53 was transcriptional inactive and responsible for autophagy in human normal BJ cells and cancer HepG2 cells under curcumin treatment. Of note, CSN5-initiated cellular autophagy by curcumin treatment was abolished in p53-null HCT116p^53−/−^ cancer cells, which could be rescued by reconstitution with wild-type p53 or transcription inactive p53 mutant p53^R273H^. Furthermore, CSN5-controlled p53 conferred a pro-survival autophagy in diverse cancer cells response to curcumin. Genetic p53 deletion, as well as autophagy pharmacological inhibition by chloroquine, significantly enhanced the therapeutic effect of curcumin on cancer cells *in vitro* and *in vivo*, but not normal cells. This study identifies a novel CSN5-controlled p53 in autophagy of human cells. The p53 expression state is a useful biomarker for predicting the anticancer therapeutic effect of curcumin. Therefore, the pharmacologic autophagy manipulation may benefit the ongoing anticancer clinical trials of curcumin.

## INTRODUCTION

Cancer is a leading cause of death around the world. CSN5 is a dominant component of the cytoplasmic COP9 signalosome (CSN) protein complex [1, 2]. CSN5 is over-expressed in different human cancers [1, 3]. Tumor suppressor p53 is an inducible master transcriptional activator. Its induction displays potent cellular growth inhibition and/or apoptosis, greatly contributing to tumor repression as well as therapeutic effects of anticancer drugs [4–6]. CSN5 can sequester p53 at the cytoplasm by either directly binding to promote the protein into the ubiquitin-proteasome-mediated protein degradation, or by linking the protein to the COP9 complex [7, 8]. Silencing CSN5 triggers cancer cell apoptosis with significant up-regulation of p53 protein [7, 8]. Thus, p53 inhibition is a proposed role of CSN5 in cancers [1, 3]. Natural plant compound curcumin selectively kills cancer cells without damaging the normal cells [9–11]. In fact, CSN5 is known as a specific target down-regulated by curcumin [1, 12]. Curcumin induces cancer cell apoptosis concomitantly with CSN5 down-regulation and p53 accumulation, underlying its anticancer activity [1, 8].

The roles of p53 in cancer therapeutic responses have become complex for its involvement in the cellular macroautophagy (briefed as autophagy) regulation [13–15]. Autophagy induction is widespread in cancer treatment response to many anticancer drugs [16, 17]. Recent studies reveal that the inducible p53 actively engages in anticancer drugs-induced autophagy [13, 18]. p53 can transactivate expression of autophagy-related genes (ATGs) [6]. Many p53 target genes encoding products, such as p21, PUMA, Bax and Bak, have the potent autophagy-initiating effects [5, 19, 20]. Therefore, p53 in autophagy makes its implication complicated in cancer therapeutic responses to different anticancer drugs [13, 21, 22]. For example, autophagy induced by many DNA damaging chemotherapeutics is important for p53-induced apoptosis in cancer cells [6]. However, the induced p53 confers a protective autophagy and fosters the cancer cell survival under the treatment of antimetabolic drug metformin [23]. Curcumin can induce autophagy, but the significance in its anticancer activity is confounded presently [24, 25]. Notably, curcumin provokes autophagy in cancer cells within 6 h, a process significant faster than other drugs in the dynamics [16, 22, 25]. Of note, curcumin, but not many other anticancer drugs, induces p53 predominantly by promoting CSN5 degradation [1, 7]. Whether and what a cause and effect relationship between CSN5-controlled p53 and autophagy induction in human cellular response to curcumin has not been addressed yet.

In this study, we demonstrated that targeting CSN5 turned on an uncorrelated transcription action of p53 for rapidly mediating autophagy in both human normal and cancer cells in response to curcumin treatment, and that constituted a protective mechanism in diverse cancer cells for survival during and after curcumin treatment. This finding thus identified a novel unique CSN5/p53-induced autophagy pathway in human cells, and a unified human cellular responding mechanism to curcumin-induced autophagy, with curcumin implication for the currently undergoing anticancer clinical trials.

## RESULTS

### Dynamics and property of curcumin-induced p53 by targeting CSN5

**Figure 1:**
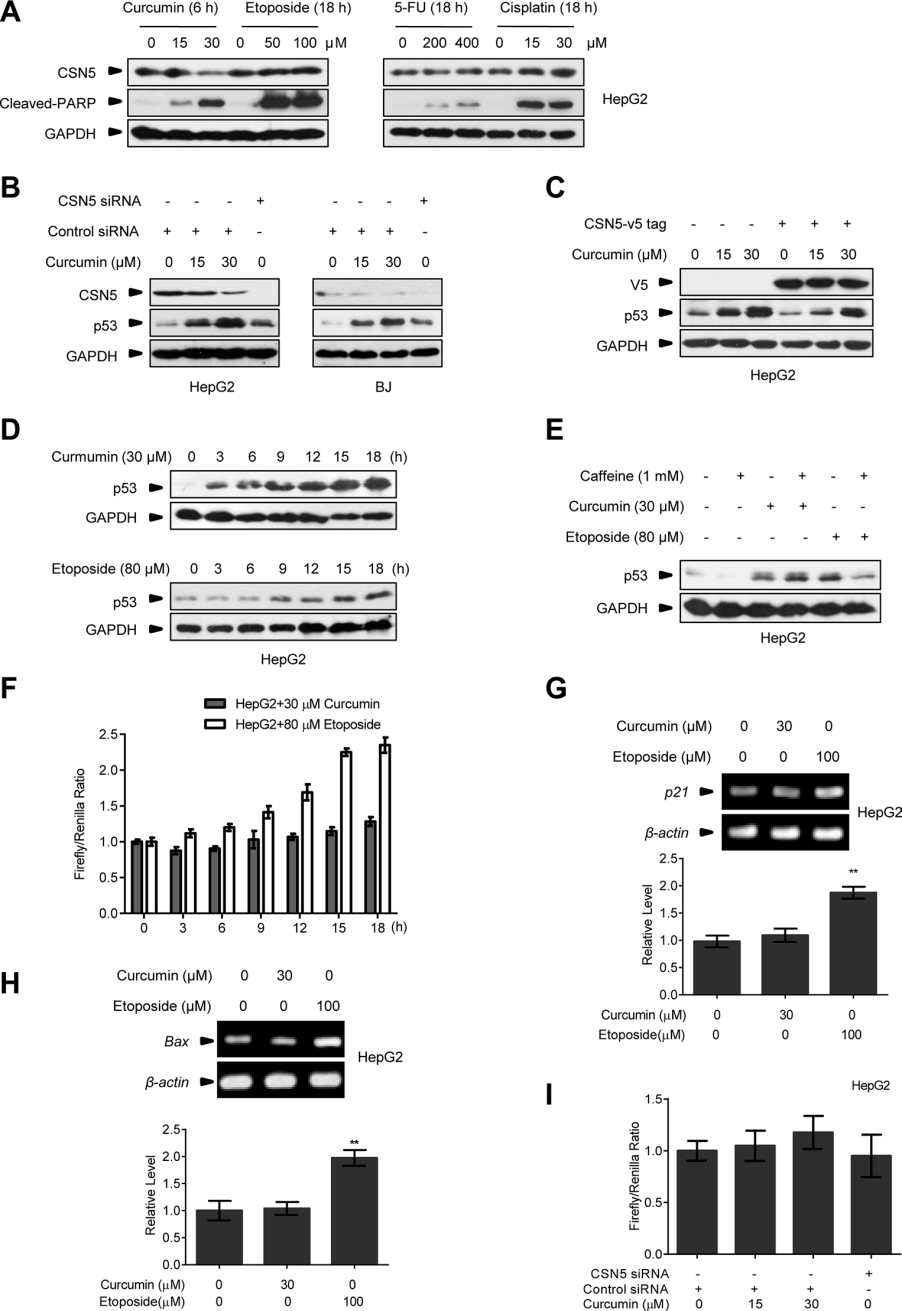
The effect of curcumin on CSN5 and p53 (**A**) Representative Western blot images in HepG2 cells treated with curcumin for 6 h, etoposide for 12 h, 5-FU for 12 h or cisplatin for 12 h. (**B**) Representative Western blot images in HepG2 and BJ cells pre-transfected with CSN5 siRNA or control siRNA for 48 h, and then treated with curcumin for 6 h. (**C**) Representative Western blot images in HepG2 cells pre-infected with lentivirus expressing JAB1-V5 tag fusion, and then treated with curcumin for 6 h. (**D**) Representative Western blot images in HepG2 cells treated with curcumin or etoposide for 3, 6, 9, 12, 15 or 18 h, respectively. (**E**) Representative Western blot images in HepG2 cells pre-treated with caffeine for 6 h, and then treated with curcumin for 6 h or etoposide for 12 h, respectively. (**F**) p53 transcriptional activity was detected by luciferase reporter assay in HepG2 cells treated with curcumin or etoposide for 3, 6, 9, 12, 15 or 18 h. (**G**) RT-PCR analysis of p21 expression levels in HepG2 cells treated with curcumin or etoposide for 12 h. Data were the mean value of 3 independent experiments. Values are expressed as the mean ± SEM, *n* = 3, ^*^p < 0.05, ^**^p < 0.01 *vs.* control group. (**H**) RT-PCR analysis of Bax expression levels in HepG2 cells treated with curcumin or etoposide for 12 h. Data were the mean value of 3 independent experiments. Values are expressed as the mean ± SEM, *n* = 3, ^*^p < 0.05, ^**^p < 0.01 *vs.* control group. (**I**) p53 transcriptional activity was detected in HepG2 cells pre-transfected with CSN5 siRNA or control siRNA for 48 h, and then treated with curcumin for 6 h.

We found that curcumin conferred a rapid down-regulating effect on CSN5 protein expression at 6 h post-treatment in human hepatic carcinoma HepG2 cells in the dose-dependent manner, but CSN5 down-regulation was not observed for other anticancer drugs including etoposide, fluorouracil (5-FU) and cisplatin in the same cells even for long time exposure (18 h) (Figure 1A). The rapid CSN5 down-regulation effect by curcumin was also repeated in the primary human normal BJ fibroblasts, and as expected, CSN5 down-regulation concomitantly with significant accumulation of p53 protein had the same dynamics in both BJ and HepG2 cells (Figure 1B). Notably, transfection with a designed CSN5 siRNA, which caused endogenous CSN5 depletion, did not give the comparable p53 accumulation with curcumin (at 30 μM dosage most manifesting) in both HepG2 and BJ cells (Figure 1B). We interpret this due to that, in addition to CSN5 down-regulation, curcumin also inhibits the COP9-related kinase activity that affect p53 instability [7]. Consistently, ectopic over-expression of a V5-tagged CSN5 (CSN5-V5), thereby overcoming cellular CSN5 depletion by curcumin, was able to significantly but not completely repress p53 induction by 15 and 30 μM curcumin treatment in HepG2 cells (Figure 1C). Further manifesting the characteristic for curcumin-induced p53 reaction, p53 induction dynamics for the DNA-damaging etoposide (80 μM) was obviously delayed (with the significant induction for 9 h- *v.s* 3 h-treatment of 30 μM curcumin, Figure 1D). Incubation with DNA damage responding kinases ATR/ATM inhibitor caffeine [26] (1 mM) was able to block etoposide- but not curcumin-induced p53 reaction in HepG2 cells (Figure 1E). Most remarkably, in HepG2 cells previously transfected with a p53-responding element-controlled luciferase reporter gene expressing plasmid, 30 μM curcumin treatment did not give a significant induction of luciferase activity compared with 80 μM etoposide (Figure 1F). RT-PCR assay confirmed that a significant induction of p53 target genes p21 and Bax mRNA were present in HepG2 cells treated with 100 μM etoposide but not 30 μM curcumin (Figure 1G and 1H). Moreover, CSN5 siRNA silencing had no effects on the induction of the transfected reporter plasmid encoded luciferase activity in HepG2 cells (Figure 1I). Taken together, we propose that CSN5 down-regulation by curcumin is responsible for giving rise to a rapid p53 protein accumulation without significant activation of intrinsic transcriptional activity of this transcriptional factor, implying a special significance for CSN5-controlled p53 in human cellular response to curcumin.

### Targeting CSN5 by curcumin turns on a prompt autophagy correlated to p53 but being dispensable for its transcriptional activity

**Figure 2:**
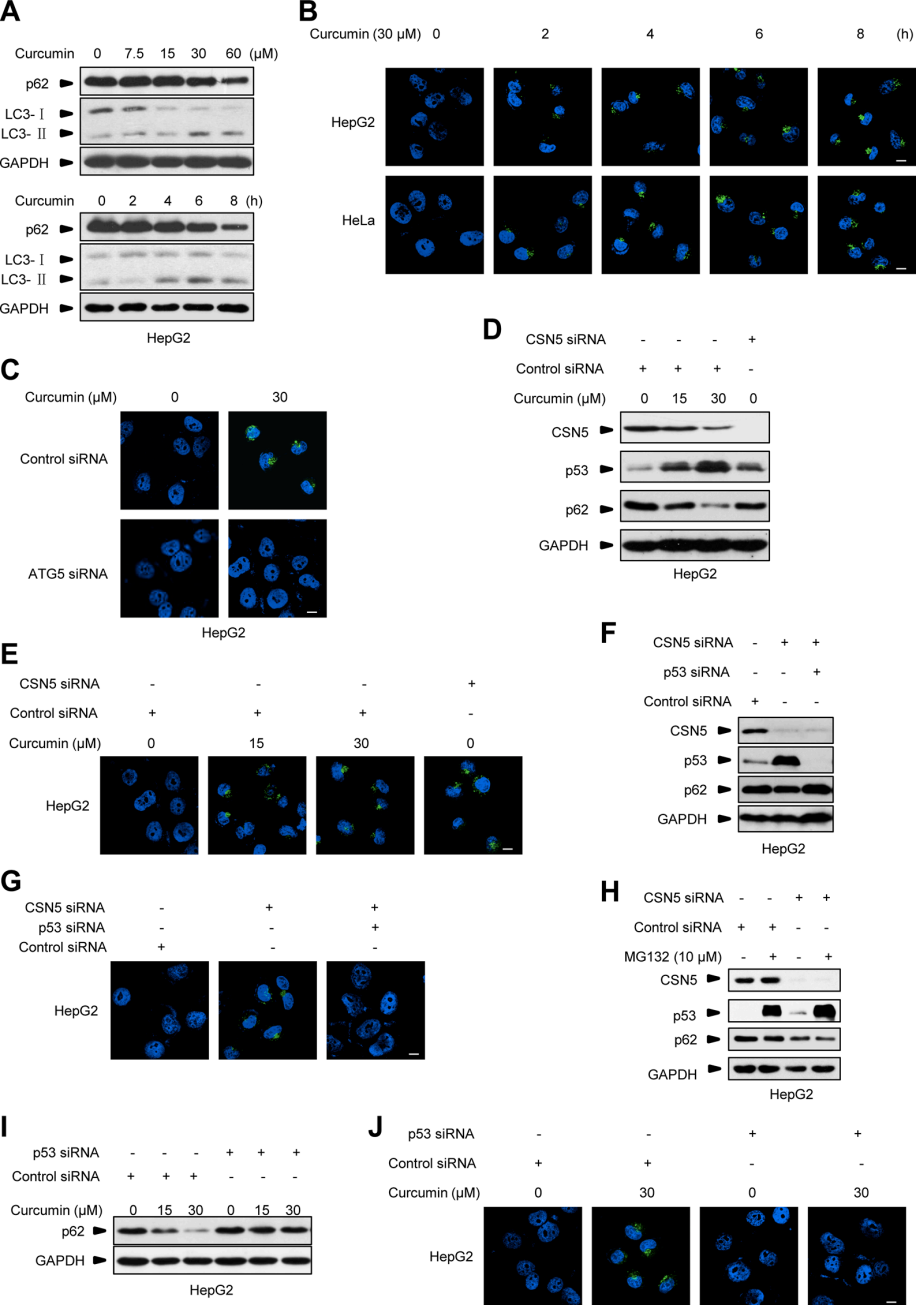
Curcumin-induced autophagy correlates to p53 (**A**) Representative Western blot images in HepG2 cells treated with 30 μM curcumin for 2, 4, 6 and 8 h, or 7.5, 15, 30 and 60 μM curcumin for 6 h. (**B**) Representative images of autophagy detection in HepG2 and HeLa cells treated with curcumin for 2, 4, 6 and 8 h. (**C**) Representative images of autophagy detection in HepG2 cells pre-transfected with ATG5 siRNA or control siRNA for 48 h, and then treated with curcumin for 6 h. (**D**) Representative Western blot images in HepG2 cells pre-transfected with CSN5 siRNA or control siRNA for 48 h, and then treated with curcumin for 6 h. (**E**) Representative images of autophagy detection in (D) were showed. (**F**) Representative Western blot images in HepG2 cells transfected with CSN5 siRNA or control siRNA, or combination of CSN5 and p53 siRNA for 48 h. (**G**) Representative images of autophagy detection in (F) were showed. (**H**) Representative Western blot images in HepG2 cells pre-transfected with CSN5 siRNA or control siRNA for 48 h, and then treated with 10 μM MG132 for 6 h. (**I**) Representative Western blot images in HepG2 cells pre-transfected with p53 siRNA or control siRNA for 48 h, and then treated with curcumin for 6 h. (**J**) Representative images of autophagy detection were showed in HepG2 cells pre-transfected with p53 siRNA or control siRNA for 48 h, and then treated with curcumin for 6 h, respectively. Scale bar: 10 μm.

A recent study shows that curcumin treatment elicits autophagy in treating colonic cancer cells at 6 h [25]. We further showed that inducible autophagy activation markers, such as p62 protein decrease and conversion of microtubule-associated protein 1 light chain 3 (LC3)-I to LC3-II were readily detected in 30 μM curcumin-treated HepG2 cells at 4 h (Figure 2A), consistently with the dynamics of CSN5 degradation and p53 induction under this condition (Figure 1B). By using a more sensitive fluorescent assay kit specific for the *in vivo* autophagosome formation [27], the inducible puncta fluorescence-staining signals around the nucleus were detected in the two different cancer cells HepG2 and cervical carcinoma HeLa cells at as early as 2 h post-30 μM curcumin treatment (Figure 2B). These inducible fluorescent signals were able to be diminished by pre-transfection with a specific siRNA against the autophagy essential gene ATG5 [13] 48 h before curcumin treatment (Figure 2C, with HepG2 cells as representative). Moreover, CSN5 siRNA also induced autophagosome formation in HepG2 cells (Figure 2D and 2E). However, when CSN5 siRNA was co-transfected with a p53 specific siRNA, thereby blocking CSN5 siRNA-induced p53 accumulation, also diminished the inducible p62 degradation (Figure 2F) and autophagosome formation (Figure 2G) in HepG2 cells. MG132 is a typical proteasome inhibitor. The treatment of MG132 alone for 6 h didn't cause p62 degradation in HepG2 cells, but induced p62 degradation in HepG2 cells pre-transfected with CSN5 siRNA (Figure 2H), further indicating that the accumulation of p53 through an inhibition of its degradation may not enhance autophagy in the presence or absence of CSN5 siRNA. Of note, pre-transfection with p53 siRNA (48 h before) ablated curcumin-induced p62 degradation and autophagosome formation in HepG2 cells (Figure 2I and 2J). These results suggest that curcumin raises a rapid autophagy induction through CSN5-controlled p53 action in human cancer cells.

**Figure 3:**
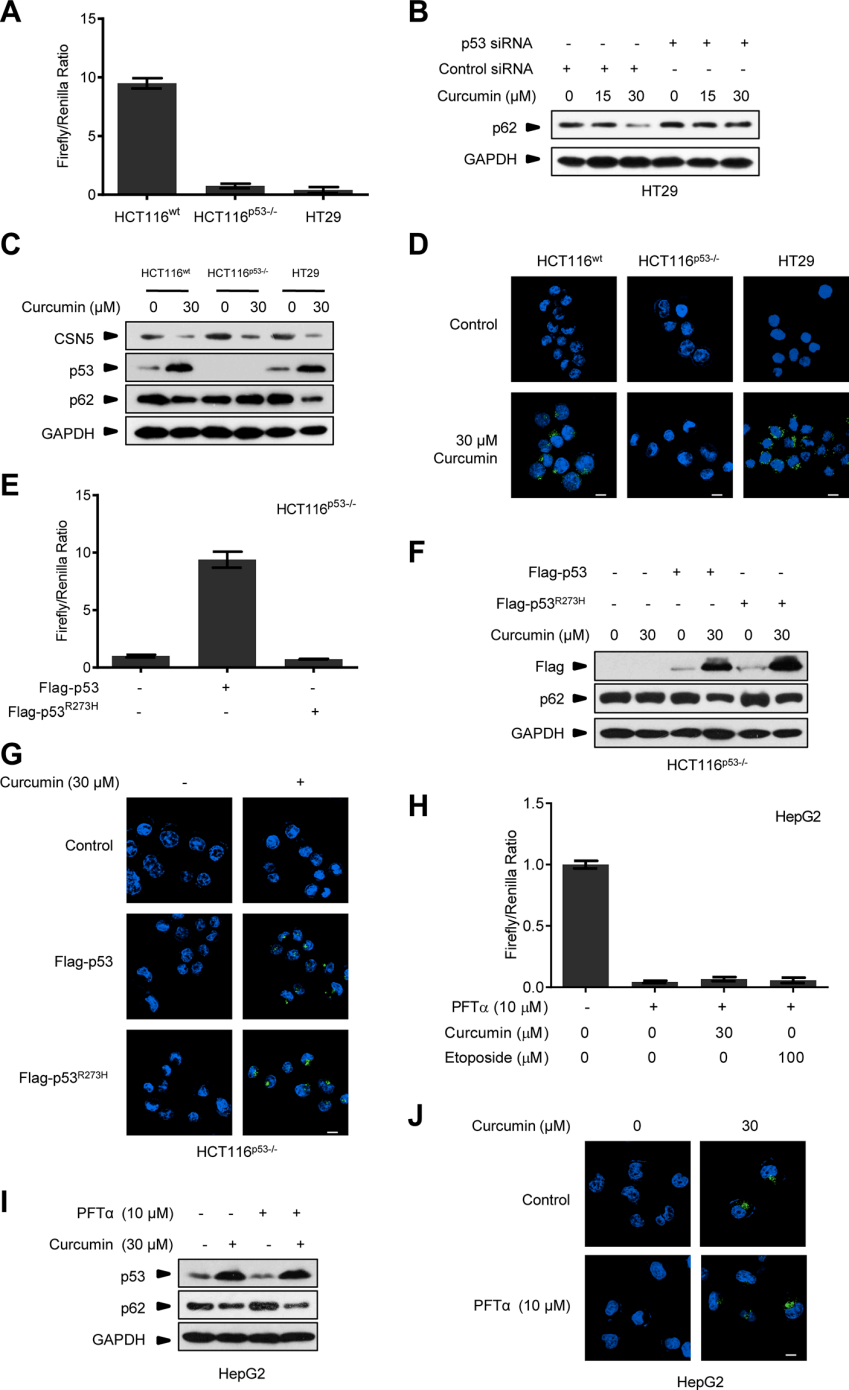
Curcumin controls p53 to induce autophagy uncorrelated to its transcriptional activity (**A**) Transcriptional activity analysis of p53 in HCT116^wt^, HCT116^p53−/−^ and HT29 cancer cells. (**B**) Representative Western blot images in HT29 cells pre-transfected with p53 siRNA or control siRNA for 48 h, and then treated with curcumin for 6 h. (**C**) Representative Western blot images in HCT116^wt^, HCT116^p53−/−^ and HT29 cells treated with curcumin for 6 h. (**D**) Representative images of autophagy detection were showed in HCT116^wt^, HCT116^p53−/−^ and HT29 cells treated with curcumin for 6 h. (**E**) Transcriptional activity analysis of p53 in HCT116^p53−/−^ cancer cells infected with lentivirus expressing flag-p53 or flag-p53^R273H^ tag fusion. (**F**) Representative Western blot images in HCT116^p53−/−^ cells pre-infected with lentivirus expressing flag-p53 and flag-p53^R273H^ tag fusion, and then treated with curcumin for 6 h. (**G**) Representative images of autophagy detection in (F) were showed. (**H**) Transcriptional activity of p53 in HepG2 cells pre-treated with PFTα for 6 h and then treated with curcumin for 6 h or etoposide for 12 h. (**I**) Representative Western blot images in HepG2 cells pre-treated with PFTα for 6 h, and then treated with curcumin for 6 h. (**J**) Representative images of autophagy detection in (I) were showed. Scale bar: 10 μm.

Furthermore, we used the pair of HCT116^wt^ and HCT116^p53−/−^ human colonic cancer cells, as well as HT29 human colonic cancer cells which have an R273H mutation in p53 and lost its transcriptional activity (Figure 3A). As shown in Figure 3B, pre-transfection with p53 siRNA (48 h before) blocked curcumin-induced p62 degradation in HT29 cells. Curcumin treatment triggered CSN5 degradation, p53 accumulation, and p62 down-regulation concomitantly at 6 h in HCT116^wt^ and HT29 cells, respectively, but the same CSN5 down-regulation failed to alter p62 in p53-null HCT116^p53−/−^ cells (Figure 3C). Also, 30 μM curcumin-induced autophagosome was present in HCT116^wt^ and HT29 cells but not in the p53-null cells (Figure 3D). Moreover, p53-null HCT116^p53−/−^ cells were respectively reconstituted with the recombinant lentiviral vector-mediated wt Flag-p53 and the point mutant Flag-p53^R273H^, which constitutively lost the transcriptional activity (Figure 3E). As shown in Figure 3F and 3G, both the stable Flag-p53 and Flag-p53^R273H^ remained the same accumulation response to 30 μM curcumin treatment, accompanying with the recovery of inducible p62 down-regulation (Figure 3F) and autophagosome formation (Figure 3G) in reconstituted HCT116^p53−/−^ cells. On the other hand, incubation with chemical p53 transcriptional activity inhibitor pifithrin-α (PFTα) [28] in HepG2 cells, significantly inhibited p53 transcriptional activity under curcumin or etoposide treatment (Figure 3H), but did not change curcumin-induced p62 reduction and autophagosome formation (Figure 3I and 3J). These results suggest that CSN5-controlled p53 is responsible for triggering an acute autophagy induction in cellular response to curcumin via an action uncorrelated to its classic transcriptional factor function, revealing a novel specific CSN5-p53-autophagy pathway induced by curcumin in human cancer cells.

### CSN5-controlled p53 provides cancer cells a protective autophagy against curcumin treatment

**Figure 4:**
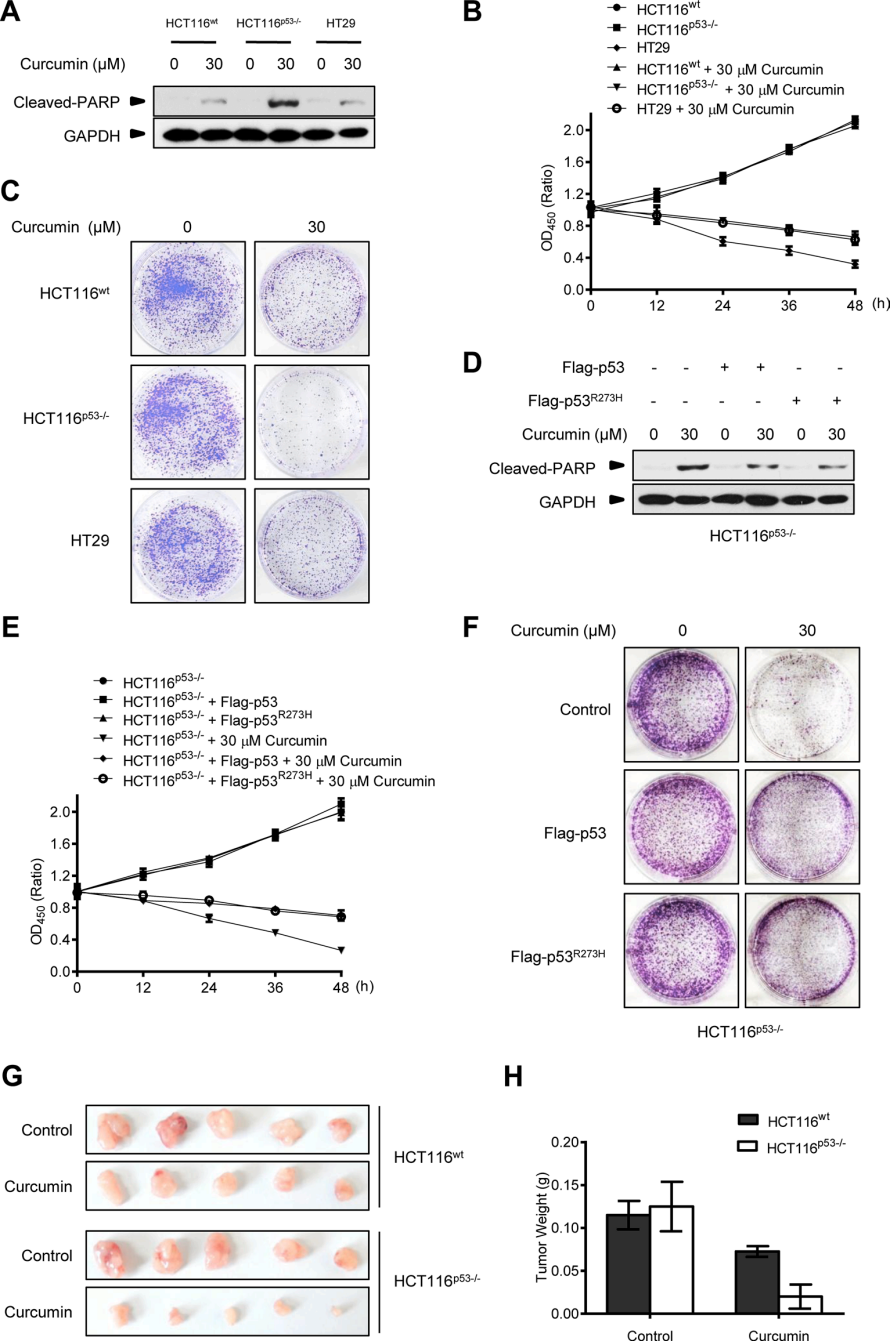
p53-controlled autophagy promotes cancer cell survival under curcumin treatment (**A**) Representative Western blot images in HCT116^wt^, HCT116^p53−/−^ and HT29 cells treated with curcumin for 6 h. (**B**) Cell number was determined by CCK-8 assay in HCT116^wt^, HCT116^p53−/−^ and HT29 cells treated with curcumin for 6 h. (**C**) The longer term effects of HCT116^wt^, HCT116^p53−/−^ and HT29 cells treated with curcumin for 6 h were assayed in relation to clonogenic survival. (**D**) Representative Western blot images in HCT116^p53−/−^ cells infected with lentivirus expressing flag-p53 or flag-p53^R273H^ tag fusion and treated with curcumin for 6 h. (**E**) Cell number was determined by CCK-8 assay in HCT116^p53−/−^ and HCT116^p53−/−^ cells pre-infected with lentivirus expressing flag-p53 or flag-p53^R273H^ tag fusion treated with curcumin for 6 h. (**F**) The longer term effects in HCT116^p53−/−^ and HCT116^p53−/−^ cells expressing flag-p53 or flag-p53^R273H^ tag fusion that treated with curcumin for 6 h were assayed in relation to clonogenic survival. (**G**) The visual comparison of tumor size from 5 mice each group of HCT116^wt^ and HCT116^p53−/−^ cells treated with control or curcumin for 6 h before injection. (**H**) Tumor weights of (G) were measured.

**Figure 5:**
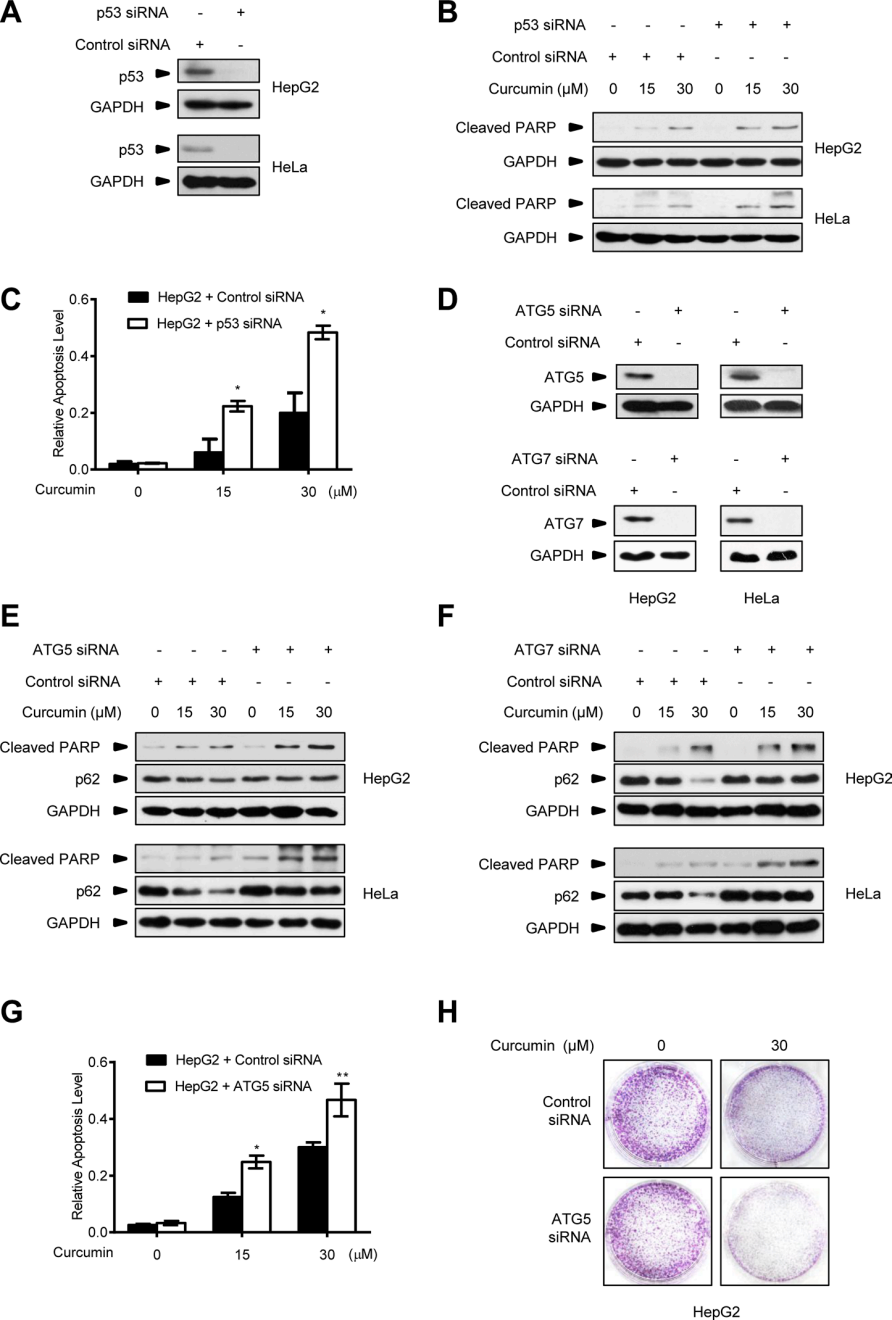
Autophagy inhibition improves the killing effect of curcumin on p53-positive cancer cells (**A**) Representative Western blot images in HepG2 and HeLa cells transfected with p53 siRNA or control siRNA for 48 h. (**B**) Representative Western blot images in HepG2 and HeLa cells pre-transfected with p53 siRNA or control siRNA for 48 h, and then treated with curcumin for 6 h. (**C**) Quantification of TUNEL-positive HepG2 cells pre-transfected with p53 siRNA or control siRNA for 48 h, and then treated with curcumin for 6 h. Data were the mean value of independent experiments with each count of no less than 100 cells. Values are expressed as the mean ± SEM, *n* = 3, ^*^p < 0.05, ^**^p < 0.01 *vs.* HepG2 cells transfected with control siRNA group. (**D**) Representative Western blot images in HepG2 and HeLa cells transfected with ATG5 siRNA, ATG7 siRNA or control siRNA for 48 h. (**E**) Representative Western blot images in HepG2 and HeLa cells pre-transfected with ATG5 siRNA or control siRNA for 48 h, then treated with curcumin for 6 h. (**F**) Representative Western blot images in HepG2 and HeLa cells pre-transfected with ATG7 siRNA or control siRNA for 48 h, then treated with curcumin for 6 h. (**G**) Quantification of TUNEL- positive HepG2 cells pre-transfected with ATG5 siRNA or control siRNA for 48 h, and then treated with curcumin for 6 h. Data were the mean value of the independent experiments with each count of no less than 100 cells. Values are expressed as the mean ± SEM, *n* = 3, ^*^p < 0.05, ^**^p < 0.01 *vs.* HepG2 cells transfected with control siRNA group. (**H**) The longer term effects of HepG2 cells pre-transfected with ATG5 siRNA or control siRNA for 48 h and then treated with curcumin for 6 h were assayed in relation to clonogenic survival.

Meanwhile, we repeatedly found that p53-null HCT116^p53−/−^ cancer cells were much more sensitive to the killing effect of curcumin than its wt counterpart and HT29 cells. As shown in Figure 4A, after 6 h of 30 μM curcumin treatment, the induction levels of the cleaved-PARP, an established molecular marker for cellular apoptotic response [29], were significantly higher in HCT116^p53−/−^ cells than in HCT116^wt^ and HT29 cells. Cell counting kit-8 (CCK-8) analysis showed that less living cells existed in HCT116^p53−/−^ culture compared with HCT116^wt^ and HT29 cells at each indicated time-point under the same curcumin treatment (Figure 4B). 15-day clonogenic assay showed that remarkable fewer living clones were remained in HCT116^p53−/−^ culture compared with HCT116^wt^ and HT29 culture after a single dose (30 μM) of curcumin treatment for 6 h (Figure 4C), respectively. Consistently, pre-expression of either Flag-p53 or Flag-p53^R273H^ mutant was able to significantly attenuate the effect of curcumin on cleaved-PARP induction (Figure 4D), and significantly enhanced both the short-termed (CCK-8 assay, Figure 4E) and long-termed (clonogenic assay, Figure 4F) survival of HCT116^p53−/−^ cells during and after 30 μM curcumin treatment. Furthermore, 30 μM curcumin treatment for 6 h in cultures significantly impaired tumorigenicity of HCT116^p53−/−^ cells in the Nude mouse xenograft model, but only modestly impaired tumorigenicity for HCT116^wt^ cells by the same *in vivo* assay (Figure 4G and 4H). Correspondingly, silencing endogenous p53 by specific siRNA (Figure 5A) significantly sensitized the two different HepG2 and HeLa cancer cells to curcumin-induced apoptosis (Figure 5B) and cell death (Figure 5C). Resembling to the effect of p53 siRNA, pre-transfection of ATG5 siRNA or ATG7 siRNA [13], significantly up-regulated cleaved-PARP protein levels in HepG2 and HeLa cells (Figure 5D). ATG5 siRNA also significantly reduced both the short-termed (terminal eoxynucleotidyl transferase dUTP nick end labeling (TUNEL) assay) and long-termed (colongenic assay) survival of HepG2 cells after 6 h treatment of 30 μM curcumin (Figure 5G and 5H). These results suggest that CSN5-controlled p53 confers a protective autophagy in cancer cells for survival from the killing effect of curcumin. Therefore, p53 genetic deletion, similar to autophagy interruption, both greatly impairs cancer cell survival under curcumin treatment.

### Autophagy inhibitor benefits the therapeutic effect of curcumin on p53-positive cancers

**Figure 6:**
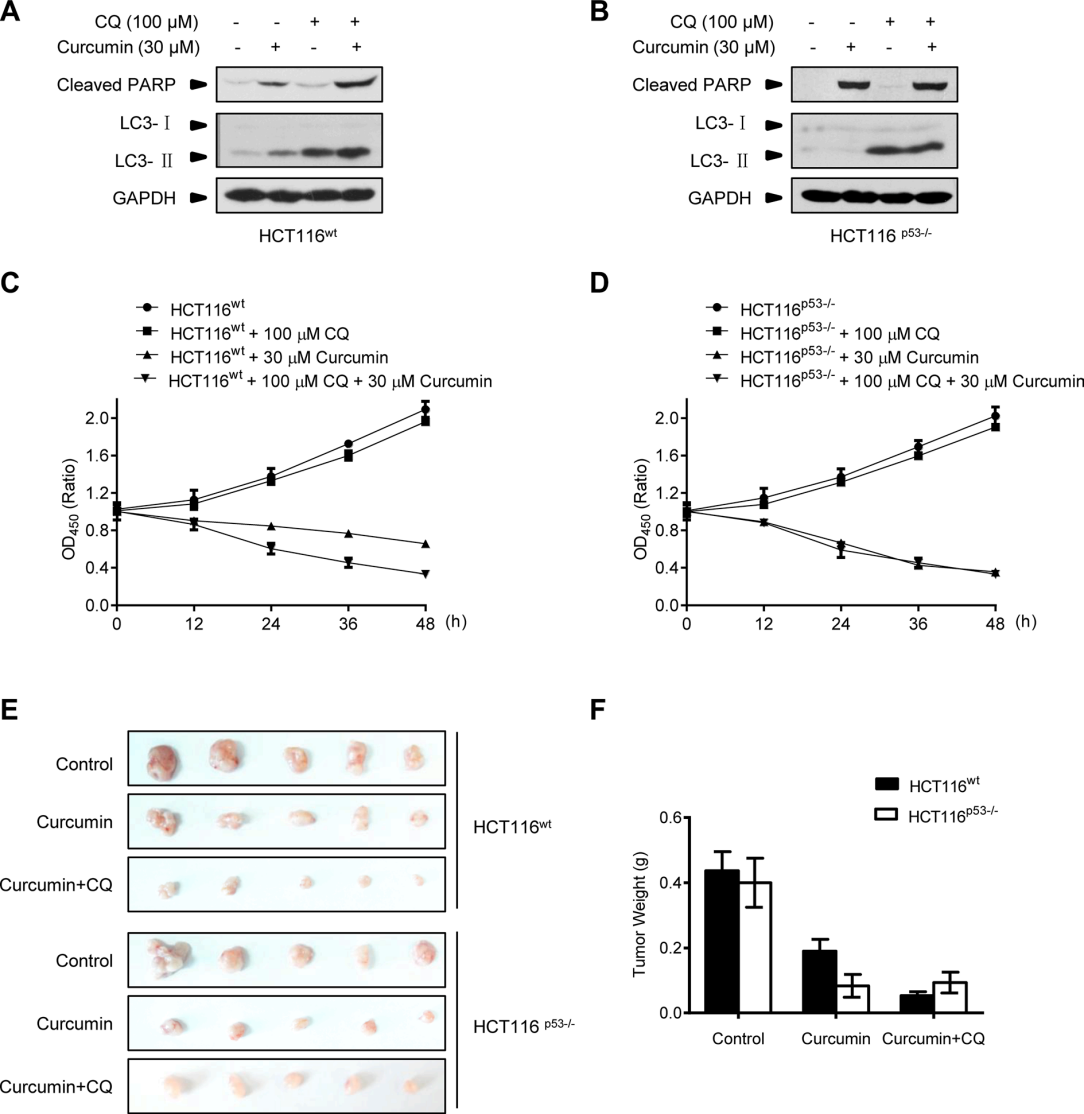
Combination with lysosome inhibitor improves the killing effect of curcumin in Nude-mice xenograft p53-positive HCT116 tumors (**A** and **B**) Representative Western blot images in HCT116^wt^ and HCT116^p53−/−^ cells pre-treated with CQ for 2 h, and then treated with curcumin for 6 h. (**C** and **D**) Cell number was determined by CCK-8 assay in HCT116^wt^ and HCT116^p53−/−^ cells treated with CQ for 2 h, and then curcumin for 6 h. (**E**) Mice bearing HCT116^wt^ or HCT116^p53−/−^ tumors were injected every 2 days with either: 0.01% DMSO (Control), 100 μM curcumin or combination of 100 μM curcumin and 100 μM CQ for 3 weeks. The visual comparison of tumor size from 5 mice of each group was showed. (**F**) Tumor weights in (E) were measured.

Pharmacologic autophagy inhibitors have been considered for clinical use to improve the effects of current anticancer therapeutics, limited by the difficulty in the predication of autophagy induction and significance for different drugs to treat cancers in patients [30, 31]. The finding above that p53 expression is a determinant for curcumin-induced autophagy in diverse cancer cells thus provides a useful biomarker for autophagy manipulation in the anticancer therapy of curcumin. Therefore, we predict that pharmacologic autophagy inhibitor will benefit the therapeutic effect of curcumin on p53-positive cancers. To test this, the proved lysosome/autophagy inhibitor chloroquine (CQ) was used to evaluate its combination on the therapeutic effect of curcumin in HCT116^wt^ and HCT116^p53−/−^ cells both in *in vitro* and *in vivo*. As shown in Figure 6A and 6B, 100 μM CQ alone was able to accumulate LC3-II in HCT116^wt^ and HCT116^p53−/−^ cells, demonstrating its efficacy in endogenous autophagy inhibition by blocking lysosome function. Furthermore, CQ alone did not induce cleaved-PARP protein levels, and its combination with curcumin significantly increased the induction in HCT116^wt^ cells but not in p53-null HCT116^p53−/−^ cells. CCK-8 assay showed that CQ combination significantly enhanced the cytotoxic effect of curcumin on HCT116^wt^ cells but not HCT116^p53−/−^ cells (Figure 6C and 6D). We further detected the action of CQ combination on the therapeutic effect of curcumin in the Nude-mice xenograft tumors formed by HCT116^wt^ and HCT116^p53−/−^ cancer cells, respectively. After one week of the cancer cell xenografted, the indicated regimes were directly injected into the grown xenograft tumors every 2 days for 3 weeks. Consistently, both curcumin alone or in combination with CQ got the same significant therapeutic effect on HCT116^p53−/−^ tumors, without significant difference (Figure 6E). However, curcumin alone only modestly inhibited the growth of HCT116^wt^ tumors, while, combination with CQ reached to the significant inhibition effect on these p53-positive tumors compared with p53-negative tumors (Figure 6E and 6F). Therefore, genetic p53-deleted cancers are inherently sensitive to curcumin treatment without the need of additional autophagy manipulation. Thus, pharmacological autophagy inhibition benefits the therapeutic effect of curcumin on p53-positive cancers.

### Autophagy inhibition does not sensitize normal cells under curcumin treatment

**Figure 7:**
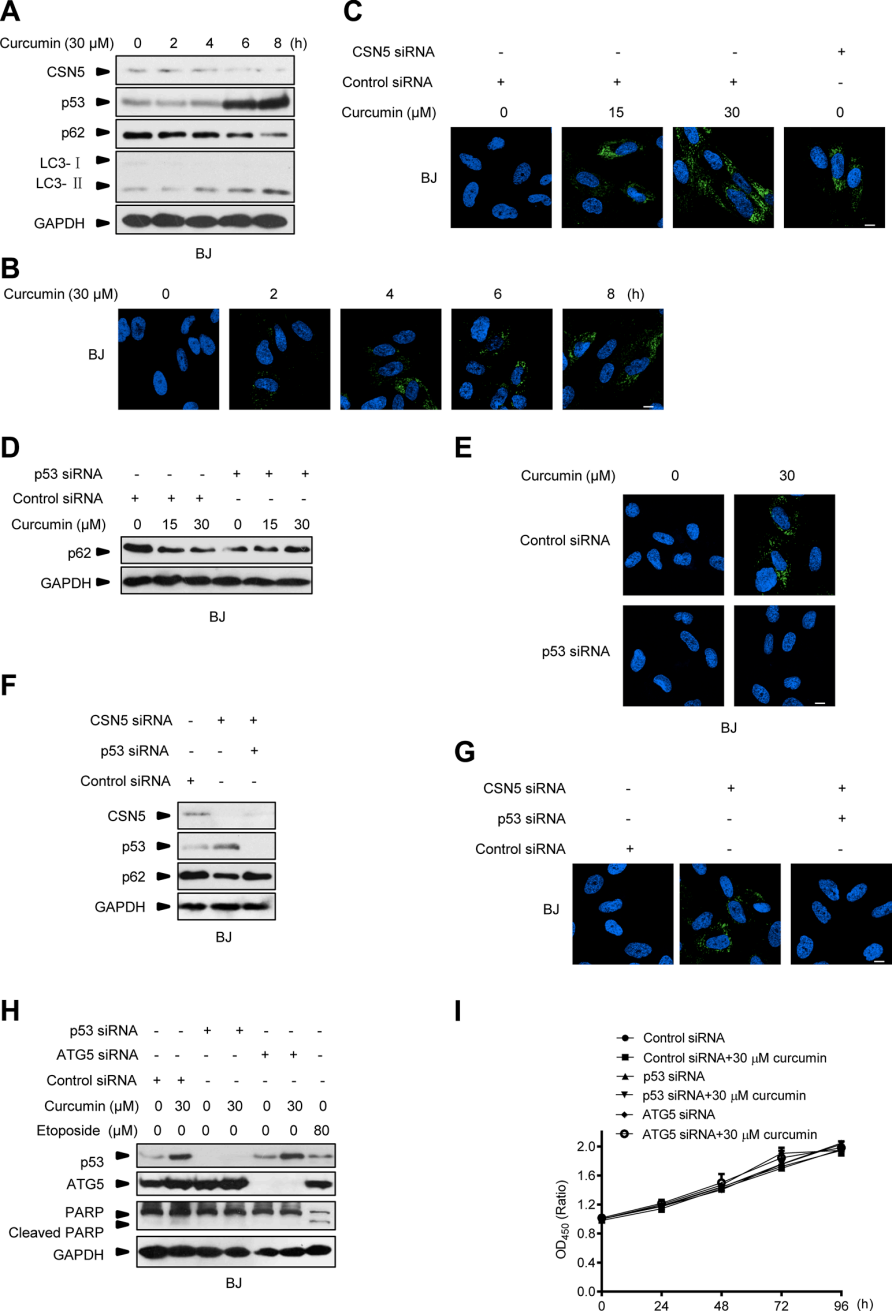
Interruption of CSN5/p53-induced autophagy pathway does not sensitize normal cells to curcumin-induced apoptosis (**A**) Representative Western blot images in BJ cells treated with curcumin for 2, 4, 6 and 8 h. (**B**) Representative images of autophagy detection in BJ cells treated with curcumin for 2, 4, 6 and 8 h. (**C**) Representative images of autophagy detection in BJ cells pre-transfected with CSN5 siRNA or control siRNA for 48 h, and then treated with curcumin for 6 h. (**D**) Representative Western blot images in BJ cells pre-transfected with p53 siRNA or control siRNA for 48 h, and then treated with curcumin for 6 h. (**E**) Representative images of autophagy detection were showed in BJ cells pre-transfected with p53 siRNA or control siRNA for 48 h, and then treated with curcumin for 6 h. (**F**) Representative Western blot images in BJ cells transfected with CSN5 siRNA or control siRNA, or combination of CSN5 and p53 siRNA for 48 h. (**G**) Representative images of autophagy detection in (F) were showed. (**H**) Representative Western blot images in BJ cells pre-transfected with p53 or ATG5 siRNA or control siRNA for 48 h, then treated with curcumin for 6 h or etoposide for 12 h. (**I**) Cell number was determined by CCK-8 assay in BJ cells transfected with control, p53 or ATG5 siRNA for 48 h, and then treated with curcumin for 6 h. Scale bar: 10 μm.

Note that the above observations were obtained in cancer cell types, we also repeated these experiments in the normal primary BJ cells. As expected, 30 μM curcumin treatment for 3 h elicited the response of CSN5 down-regulation, p53 accumulation, p62 degradation and LC3-I to LC3-II conversion, as well as inducible autophagosome formation in BJ cells (Figure 7A and 7B). Endogenous CSN5 RNAi also induced autophagosome formation in these normal cells (Figure 7C). Furthermore, p53 RNAi silencing was also able to block curcumin- or CSN5 siRNA-induced autophagosome formation in BJ cells (Figure 7D–7G). These results thus indicate that CSN5-p53-autophagy pathway is a unifying responding mechanism in curcumin-treated human normal cells. But unlike the observations in cancer cells, interruption of this pathway by either ATG5 or p53 siRNA did not sensitize normal BJ cells to curcumin-induced PARP cleavage apoptotic response (Figure 7H). Both ATG5 siRNA- and p53 siRNA-transfected BJ cells remained the same living activity as the parental BJ cells under 30 μM curcumin treatment (Figure 7I). These findings also highlight that autophagy manipulation is safety in improving the anticancer therapy of curcumin.

## DISCUSSION

Curcumin has shown promise as a safe and specific anticancer agent for selectively killing cancer cells, but not normal cells [9, 10]. CSN5 controls p53 stability by increasing its degradation [7]. p53 actively engages in anticancer drugs-induced autophagy [21]. Curcumin selectively represses diverse cellular oncoprotein expression and up-regulates p53 [9, 32, 33]. CSN5 is a known specific target for curcumin [3, 8]. Our results demonstrated that CSN5 down-regulation by CSN5 siRNA or curcumin triggered significant p53 protein expression in cancer cells. Interestingly, curcumin induced autophagy in WT p53 or transcriptional inactive p53 cancer cells, but not p53-null cancer cells, indicating curcumin-induced autopahgy correlated to p53, but not its transcriptional activity. More importantly, p53 up-regulation was found to potently weaken anticancer activity of curcumin by a protective autophagy induction in diverse cancer cells, fostering the cancer cell survival during and after the treatment. Therefore, these results indicate that CSN5-controlled p53 may drive a pro-survival autophagy in diverse cancer cells response to curcumin.

**Figure 8:**
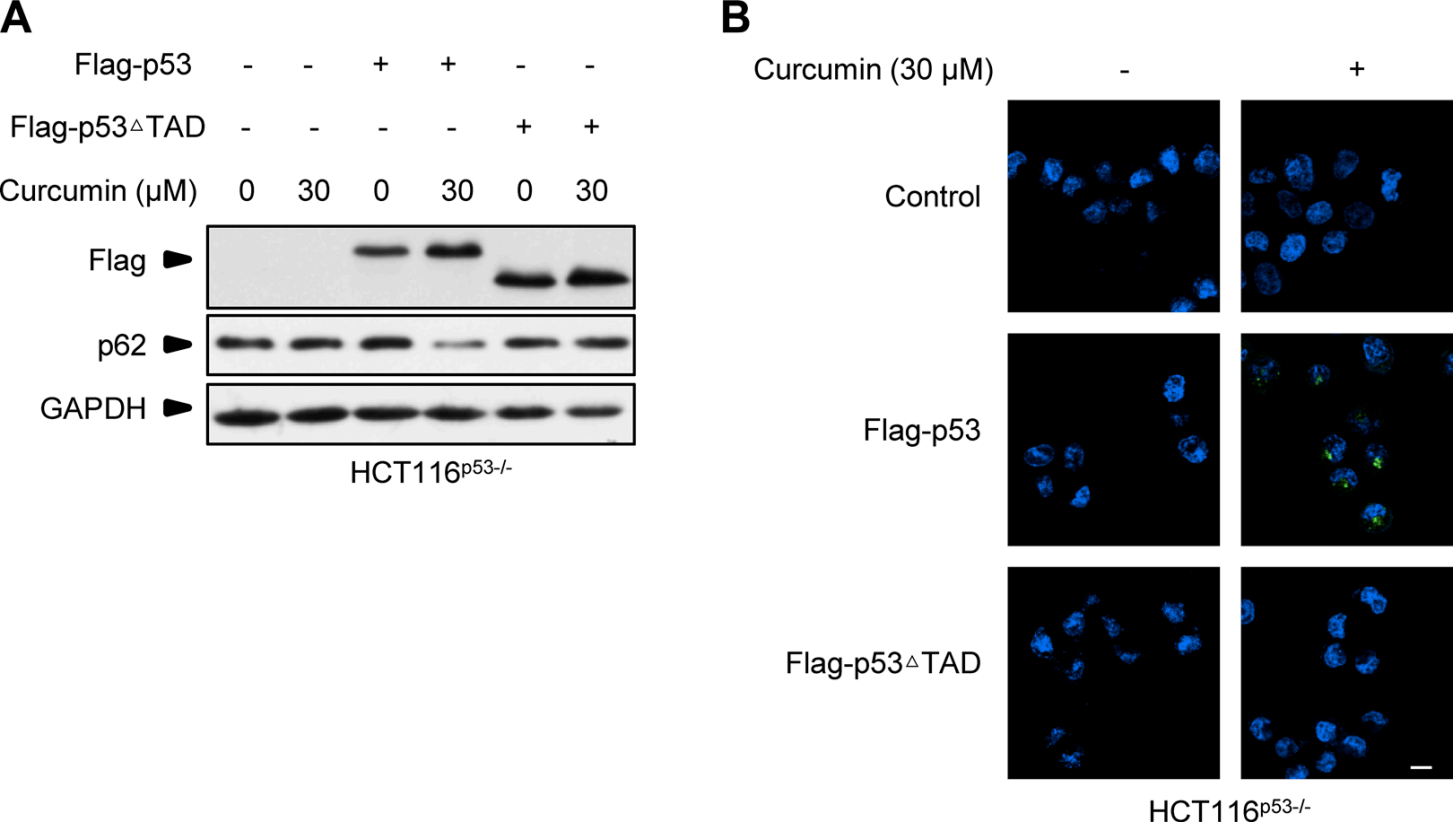
Flag-p53ΔTAD failed to recover curcumin-induced autophagy response in p53-null HCT116 cells (**A**) Representative Western blot images in HCT116^p53−/−^ cells pre-infected with lentivirus expressing flag-p53 and flag-p53**Δ**TAD tag fusion, and then treated with curcumin for 6 h. (**B**) Representative images of autophagy detection in (A) were showed.

It is known that p53 is involved in autophagy induced by many different anticancer drugs [21, 22]. But unlike these cases wherein p53 engages in the process predominantly relying on its classic transcriptional factor function regardless of the different inducing mechanisms [6, 19], curcumin-induced p53 here conducted autophagy activation by the action uncorrelated to its DNA-binding and transcriptional activity, but correlated to curcumin-binding CSN5 inducible degradation. Consistently, we found that p53 induced by curcumin or CSN5 RNAi was lack of obvious transcriptional activity. Also, although both the wt and the transcriptional activity-negative p53^R273H^ mutant were able to reconstitute autophagy induction responsibility of p53-null HCT116 cells to curcumin treatment, we found that simple ectopic over-expression of these two p53 alleles, thereby mimicking a boost accumulation of p53 protein, was unable to trigger a detectable autophagy activation in p53-null HCT116 cells, unless CSN5 siRNA was co-transfected to silence endogenous CSN5 (data not shown). Further highlighting that a signal from the previous binding governor CSN5 is important to the subsequent autophagy activation action of the controlled p53 in cellular response to curcumin, reconstitution of another p53 mutant Flag-p53**Δ**TAD, which deletes the reported N-terminal CSN5-binding-site of wt p53 [7], failed to recover curcumin-induced autophagy in the reconstituted p53-null HCT116 cells (Figure 8A and 8B). We therefore prefer to suggest that CSN5-controled p53 not only functions as a responding module for curcumin to turn on autophagy, but also pre-setting a dictation for p53 to conduct an uncorrelated action of transcription factor on autophagy activation in the cytosol, thereby accomplishing a rapid autophagy induction in human cellular response to curcumin.

In summary, this study identified a novel CSN5/p53-induced autophagy pathway inherent in human cells, with the significance for promoting a rapid autophagy response upon curcumin treatment. Since a unified pro-survival autophagy correlated to p53 protein conferred in diverse human cancer cells, this finding also provides the p53 expression state as a useful biomarker for predicting the anticancer therapeutic effect of curcumin. As compromise of autophagy induction didn't affect normal cells but greatly improved the therapeutic effect of curcumin on p53-positive cancers, this study suggests that pharmacologic autophagy manipulation will benefit the ongoing anticancer clinical trials of curcumin.

## MATERIALS AND METHODS

### Reagents and plasmids

Curcumin, etoposide, caffeine, CQ, 5-FU and cisplatin were obtained from Sigma (St Louis, MO, USA). PFTα and MG132 were from Selleck (Houston, TX, USA). Curcumin, etoposide, PFTα, rapamycin or 5-FU was dissolved in 100% dimethylsulfoxide (DMSO) to get a stock solution of 100, 50, 10, 10 or 200 mM concentrations, respectively. Caffeine, cisplatin or CQ was dissolved in deionized water to get a stock of 100, 15 or 50 mM concentrations, respectively. p53 and CSN5 cDNA were obtained by reverse transcription-PCR from total RNA of HepG2 cells. Then p53 cDNA and CSN5 cDNA were cloned into a pCDH-based lentiviral vector (expressed as flag-p53 and CSN5-V5 tag fusion protein). Flag-p53 plasmid was used as template to generate plasmid expressing flag-p53^R273H^ mutant, in which the indicated residue arginine was replaced by histidine and flag-p53ΔTAD mutant, which deleted 1–50 amino acids in N-terminal.

### Cell culture, transfection and treatment

HepG2 and HeLa cells were purchased from Cell Bank of China Academy of Medical Sciences (Beijing, China). BJ and HT29 cells were purchased from American Tissue Culture Collection (ATCC, Manassas, VA, USA). HCT116^wt^ and HCT116^p53−/−^ human colon cancer cells were a gift from National Center of Biomedical Analysis (Beijing, China). HT29 cells were grown in McCoy's 5A medium (Gibco, Carlsbad, CA, USA) supplemented with 10% FCS (Gibco). The other cells were grown in DMEM (Gibco) supplemented with 10% FCS (Gibco). All cells were cultured in a 37°C incubator with 5% CO_2_. Lipofectamine 2000 (Invitrogen, Carlsbad, CA, USA) was used for all DNA constructs and transfection according to the manufacturer's recommendations. For transfection with siRNA (100 nM), RNAmax (Invitrogen) was used. siRNAs targeting p53, CSN5, ATG5, ATG7 and the control siRNA were purchased from Invitrogen.

The cells were treated with curcumin, etoposide, 5-FU or cisplatin of indicated concentration for indicated hours, and then processed for RNA isolation and RT-PCR, Western blot, autophagy detection, clonogenic survival, luciferase reporter assay, and CCK-8 assay, respectively.

The cells were pre-treated with either 1 mM caffeine or 10 μM PFTα for 6 h, or pretreated with 100 μM CQ for 2 h, and then treated with curcumin or etoposide of dedicated concentrations and hours. These cells were processed for autophagy detection, CCK-8 assay or luciferase reporter assay. Cells were treated with 10 μM MG132 for 6 h, and then processed for Western blot assay.

All drug concentrations were selected based on our pre-experiments and the previous reports.

### RNA isolation and RT-PCR

Total RNA was isolated by Trizol reagent (Invitrogen). RT-PCR was performed with the PrimeScript 1st Strand cDNA Synthesis kit (Takara, Dalian, China) according to the manufacturer's instructions. To detect mRNA levels of *p21*, we used the following primers: forward (5′-TCTTGTACCCTTGTGCCTCGC-3′) and reverse (5′-GCTTCCAGGACTGCAGGCTTC-3′), *Bax*: forward (5′-TTCTGACGGCAACTTCA ACTG-3′) and reverse (5′-GGAGAGGAGGCCGTCCTGGAG-3′), and *β-actin* forward (5′-AGACTTCGAGCAGGAGATGG-3′) and reverse (5′-CGGATGTC AACGTCACACTT-3′). *β-actin* was used as an internal control.

### Clonogenic survival

Exponentially growing cells (1 × 10^4^) were plated in 6-well plates and grew for 24 h. Then the cells were incubated with 30 μM curcumin for 6 h. This treatment didn't cause significant cell death. Cultures were changed every other day. After 15 days, the viable colonies were fixed with methanol and then stained with methylrosanilinium chloride.

### Cell death assay (TUNEL)

Apoptotic cells were detected using the DeadEnd^™^ Colorimetric TUNEL System (Promega, Madison, WI, USA). All steps were carried out according to the manufacturer's instructions. Briefly, HepG2 cells plated on glass coverslips were grown for 12 h and then incubated with 15 or 30 μM curcumin for 6 h. Then the cells were fixed by immersing slides in 4% paraformaldehyde solution and permeabilized by immersing the slides in 0.2% Triton. After equilibration, slices were incubated with terminal deoxynucleotidil transferase (TDT) reaction mix at 37°C for 1 h, in which biotinylated nucleotide was incorporated at the 3′-OH DNA ends and stopped by 2× standard saline citrate (SSC). Then horseradish peroxidase-labeled streptavidin (Streptavidin HRP) was bound to these biotinylated nucleotides, which were detected using peroxidase substrate, hydrogen peroxide, and the stable chromogen, diaminobenzidine (DAB). Using this procedure, apoptotic nuclei were stained dark brown.

### Western blot analysis

Total proteins were extracted from the cells lysed in RIPA buffer. Thereafter, 30 μg of cell lysate were loaded each well into 12% SDS-PAGE gels and transferred to nitrocellulose filter membrane (GE Healthcare, Munich, Germany) for 1 h at 15V. The membranes were blocked in 5% non-fat milk in TBST for 1 h at room temperature and probed with primary antibodies for 2 h at room temperature. The primary antibodies GAPDH (#3683), cleaved PARP (#9541), PARP (#9542), ATG5 (#12994) and ATG7 (#2631) were from Cell Signaling Technology (Danvers, MA, USA); p62 (PM045) and LC-3 (PM036) were from Medical & Biological Labotatories (Nagoya, Japan); flag (A8592) was from Sigma; CSN5 (ab495) and V5 (ab9116) were from Abcam (Cambridge, UK); and p53 (05-224) was from Millipore (Billerica, MA, USA). Then the membranes were incubated with HRP-conjugated antirabbit IgG (Sigma, A6154) or antimouse IgG (Sigma, A9044) for 2 h at room temperature. Peroxidase activity was detected using an enhanced chemiluminescence detection kit (ThermoFisher Scientific, Waltham, MA, USA) and developed on Kodak film.

### Autophagy detection

Autophagy in cells was detected by the Cyto-ID Autophagy detection kit from Enzo Life Sciences (New York, NY, USA). First, the cultured cells were grew on coverslips for 12 h and then treated with indicated reagents for respective hours. Removing the medium and washing the cells twice with 1 × assay buffer, the cells were incubated with green fluorescence detection reagent protected from light for 15–30 min at 37°C. Then the cells were carefully washed with 1× assay buffer, and the stained cells were analyzed by confocol microscopy (Nikon, Tokyo, Japan). DAPI was used to visualize the nuclei.

### Luciferase reporter assay

Cells were transfected in triplicate with 400 ng of p53-responsive promoter, 40 ng of thymidine kinase (TK) promoter as control. 24 h later, cells were treated with the reagents indicated for indicated hours and then lysed in luciferase lysis buffer (Vigorous, Beijing, China) according to the manufacturer's instructions. These samples were assayed for luciferase activity in a luminometer using Dual-Lucy Assay Kit (Vigorous).

### CCK-8 assay

Cells were seeded in 96-well plates at a density of 1500 cells per well. After indicated treatment, cell proliferation was determined with CCK-8 assay (Dojindo Laboratories, Osaka, Japan) according to the manufacturer's instructions. The absorbance of individual wells was determined at 450 nm. The OD value of the treatment group was normalized to the value from the untreated control group. All reactions were repeated three times.

### Animal experiments and treatment

Female BALB/c mice were purchased from the Vital River Laboratory Animal Center (Beijing, China). All *in vivo* experimental procedures were followed the Institutional Animal Care Committee at Beijing Institute of Biotechnology and the Chinese Council on Animal Care at Beijing Institute of Biotechnology.

HCT116^wt^ or HCT116^p53−/−^ cells (5 × 10^6^) were first treated with DMSO (Control) or 30 μM curcumin for 6 h. And then HCT116^wt^ or HCT116^p53−/−^ cells (5 × 10^6^) were injected subcutaneously into both flanks of BALB/c mice. Mice were sacrificed 2 weeks after injection, and tumors were removed and weighed for analysis.

HCT116^wt^ or HCT116^p53−/−^ cells (5 × 10^6^) were injected subcutaneously into both flanks of BALB/c mice. At one week post-injection, xenografts were visible on both sides. Mice, bearing tumors, were randomly divided into 3 groups (*n* = 5). Mice were injected every 2 days with either: 0.01% DMSO (100 μL, Control), 100 μM curcumin (in 100 μL 0.01% DMSO) or 100 μM curcumin combined with 100 μM CQ (in 100 μL 0.01% DMSO) for 3 weeks. Mice were sacrificed 3 weeks after initiating treatment, and tumors were removed and weighed for analysis.

### Statistical analysis

Data were expressed as the mean ± standard error of the mean (SEM) deviation. Statistical analysis was performed by a Student's *t* test. Differences were considered significant at *p* < 0.05. Figures were obtained by the Statistical Analysis System (GraphPad Prism 6, GraphPad Software Inc, La Jolla, CA, USA).
